# Supplementary material for: A High-Efficient Reinforcement Learning Approach for Dexterous Manipulation
Source: Biomimetics (Basel). 2023 Jun 16;8(2):264. doi: 10.3390/biomimetics8020264 (PMC10296228; doi:10.3390/biomimetics8020264)
Supplement: Supplementary file 1 [file biomimetics-08-00264-s001.zip › biomimetics-2387948-supplementary.pdf]

biomimetics

S1. Backpropagation

The feedback from the motion increment δa_i is computed from $t = N{:}\,0$. When $t = N$:

$$V(s,N) = l_f(s_N) \tag{1}$$

Since the loss function $l(s_t, a_t)$ is concave, if a perturbation $\delta s, \delta a$ is applied to the reference point $i(s_i, a_i)$ at $t = i$, and δa is found under δs to minimize the value difference after the perturbation. It can be considered that the current reference point belongs to the optimal trajectory. The value difference $Q(\delta s, \delta a)$ can be obtained:

$$\begin{aligned} Q(\delta s,\delta a) &= \Delta V(i) \\ &= l(s_i+\delta s, a_i+\delta a) + V\big((f(s_i+\delta s, a_i+\delta a), i+1)\big) - l(s_i,a_i) + V(f(s_i,a_i), i+1) \end{aligned} \tag{2}$$

Eq.2 can be approximated as quadratic form and expanded into:

$$\begin{aligned} Q(\delta s,\delta a) &\approx \frac{1}{2}\begin{bmatrix}1\\ \delta s\\ \delta a\end{bmatrix}^T \begin{bmatrix}0 & Q_s^T & Q_a^T\\ Q_s & Q_{ss} & Q_{sa}\\ Q_a & Q_{as} & Q_{aa}\end{bmatrix} \begin{bmatrix}1\\ \delta s\\ \delta a\end{bmatrix} = \frac{1}{2}\begin{bmatrix}1\\ \delta s\\ \delta a\end{bmatrix}^T Q_i \begin{bmatrix}1\\ \delta s\\ \delta a\end{bmatrix} \\ &= \frac{1}{2}[Q_s^T \delta s_N + Q_a^T \delta a_N + \delta s_N^T Q_s + \delta s_N^T Q_{ss}\delta s_N + \delta s_N^T Q_{sa}\delta a_N + \delta a_N^T Q_a + \delta a_N^T Q_{as}\delta s_N \\ &+ \delta a_N^T Q_{aa}\delta a_N] \end{aligned} \tag{3}$$

It can be obtained from Eq.2 and 3 that:

$$\begin{aligned} Q_s &= l_s + f_s^T V_s' \\ Q_a &= l_a + f_a^T V_s' \\ Q_{ss} &= l_{ss} + f_s^T V_{ss}' f_s + V_s' f_{ss} \\ Q_{aa} &= l_{aa} + f_a^T V_{ss}' f_a + V_s' f_{aa} \\ Q_{as} &= l_{as} + f_a^T V_{ss}' f_s + V_s' f_{as} \end{aligned} \tag{4}$$

where, $\begin{bmatrix}f_s\\ f_a\end{bmatrix}$ represents the Jacobian matrix of the system transfer equation, $\begin{bmatrix}f_{ss} & f_{sa}\\ f_{as} & f_{aa}\end{bmatrix}$ represents the Hessian matrix of the system transfer equation, $\begin{bmatrix}l_s\\ l_a\end{bmatrix}$ donates the Jacobian matrix of the loss function, $\begin{bmatrix}l_{ss} & l_{sa}\\ l_{as} & l_{aa}\end{bmatrix}$ represents the Hessian matrix of the loss function, V_s' represents the Jacobian matrix of the value function V of the system at the reference point $t = i + 1$, and V_{ss}' represents the Hessian matrix of the value function V of the system at the reference point $t = i + 1$.

According to Eq. 3, at $t = i$, when $\delta a_N = -Q_{aa}^{-1}(Q_a + Q_{as}\delta s)$, the quadratic function $Q(\delta s_N, \delta a_N)$ is minimized as:

$$\delta a^* = arg\min_{\delta a} Q(\delta s,\delta a) = -Q_{aa}^{-1}(Q_a + Q_{as}\delta s) = K_i(\delta s) + k_i \tag{5}$$

where δa^* is the input disturbance.

K_i and k_i can be expressed as:

$$K_i = -Q_{aa}^{-1} Q_{as} \tag{6}$$

$$k_i = -Q_{aa}^{-1} Q_a \tag{7}$$

Substituting δa^* into Eq. 3, we can get:

$$Q(\delta s_N, \delta a_N) = \Delta V(i) = -\frac{1}{2} Q_a Q_{aa}^{-1} Q_a \tag{8}$$

The Jacobian matrix and Hessian matrix of value function can be obtained at $t = i$:

$$V_s(i) = Q_s - Q_a Q_{aa}^{-1} Q_{as} \tag{9}$$

$$V_{ss}(i) = Q_{ss} - Q_{sa} Q_{aa}^{-1} Q_{as} \tag{10}$$

$Q_t, K_t, k_t, V_s(t)$ and $V_{ss}(t)$ in $t = N{:}\,0$ can be calculated according to the above equations, and the expression of the motion increment δa_t with respect to the state change δs_t at each time interval of $t = 0{:}\,N$ is obtained. The motion sequence is applied to the forward propagation and the motion τ_{new} is updated.

S2. Forward propagation

In the back propagation, the expression $\delta a_t = K_t(\delta s) + k_t$ of the motion increment δa_t with respect to the change of state δs_t at each time interval of $t = 0{:}\,N$ is obtained. In the forward propagation, the new system trajectory is obtained by iterating the following equation under $t = 0{:}\,N$:

$$\hat{s}_0 = s_0 \tag{11}$$

$$\hat{a}_i = a_i + k_t(i) + K_t(\hat{s}_i - s_i) \tag{12}$$

$$\hat{s}_{i+1} = f(\hat{s}_i, \hat{a}_i) \tag{13}$$

where $(s_i, a_i) \in \tau_{old}, (\hat{s}_i, \hat{a}_i) \in \tau_{new}$, and in each cycle $(\hat{s}_i, \hat{a}_i)$ is renewed to τ_{new}.

The system at the reference point $i(s_i, a_i)$ is Taylor expanded in the first order and multiple iterations are carried out to obtain the optimal motion sequence and the optimal trajectory. Since the premise of local linearization is that the system state and input cannot deviate too far from the reference point $i(s_i, a_i)$ at $t = i$:

$$\left\| \begin{bmatrix} \delta s \\ \delta a \end{bmatrix} \right\| \leq d \tag{14}$$

Therefore, a linear search coefficient α is introduced, where $0 < \alpha \leq 1$. When $\alpha = 0$ and $\hat{s}_{i+1} = f(\hat{s}_i, \hat{a}_i)$ does not change, $\hat{a}_i = a_i$ and the robotic track does not change due to the same initial state, which is $\hat{s}_0 - s_0 = 0$. By increasing the value of α, the new system trajectory is close to the old system trajectory in each iteration, then Eq. 12 can be written as follows:

$$\hat{a}_i = a_i + \alpha k_t(i) + K_t(\hat{s}_i - s_i) \tag{15}$$

At the same time, in order to improve the robustness of the control, a quadratic loss term μI is added to Eq. 12 adjusting the increase of the control input a. Moreover, additional penalties are added when the system deviates from reference points. μ is the Levenberg-Marquardt(LM) coefficient. Therefore, Eq. 12 can be written as:

$$K_i = -Q_{aa}^{-1} Q_{as} = -(l_{aa} + f_a^T (V_{ss}' + \mu I) f_a + V_s' f_{aa})^{-1} (l_{as} + f_a^T (V_{ss}' + \mu I) f_s + V_s' f_{as}) \tag{16}$$

$$k_i = -Q_{aa}^{-1} Q_a = -(l_{aa} + f_a^T (V_{ss}' + \mu I) f_a + V_s' f_{aa})^{-1} (l_a + f_a^T V_s') \tag{17}$$

Then, the modified expressions of Q_{aa} and Q_{as} are put into Eq.14 and 15 to obtain the expressions of K_i and k_i at $t = i$:

$$K_i = -Q_{aa}^{-1} Q_{as} = -(l_{aa} + f_a^T (V_{ss}' + \mu I) f_a + V_s' f_{aa})^{-1} (l_{as} + f_a^T (V_{ss}' + \mu I) f_s + V_s' f_{as}) \tag{18}$$

$$k_i = -Q_{aa}^{-1} Q_a = -(l_{aa} + f_a^T (V_{ss}' + \mu I) f_a + V_s' f_{aa})^{-1} (l_a + f_a^T V_s') \tag{19}$$

Finally, in the nonlinear system, the optimal control is calculated by successive iterations, and each iteration includes the above backward propagation and forward propagation.
